# Moveable Kidney

**Published:** 1843-10

**Authors:** 


*Moveable Kidney.*—In the "Medical and Physical Journal" for 1825, (vol. ii., p. 476) mention was made of certain cases of equivocal moveable tumours in the abdomen which had been taken for diseased ovary, but which Dr. Bailie suspected were due to an abnormal state of the kidney. He had not opportunities of verifying his suspicions by postmortem examination, but the correctness of his diagnosis has since been amply confirmed.

In 1826, Aberle published in the "Salzburg Gazette," a case of a man, thirty-six years of age, who, having long suffered disturbance of the digestive organs, began to remark a moveable tumour to the right of the umbilical region, which he could with ease remove so as to press against different parts of the abdominal parietes. The tumour gradually increased, the patient fell into a state of marasmus, and ultimately died. After death the spleen was found tuberculous; the pancreas hypertrophied; the right kidney healthy, but moveable, and capable of displacement with great facility, its vessels having become much lengthened, and its cellular envelope destitute of fat.

Since the above period Aberle has witnessed and recorded three other and similar cases. One occurred in a woman, sixty-six years of age, who had died of apoplexy, and on opening whom a solid mass was found on the right side in front of the psoas muscle, which turned out to be the kidney, pushing downwards the ascending and part of the transverse colon. The second case was that of a lady, seventy-five years old, who had long perceived the presence of the tumour, which became more moveable after a sharp attack of dysentery. She, however, suffered little or nothing from it beyond some pain after fatiguing walks. The third case occurred after menstrual disorder; but the patient suffered little material inconvenience. In The Lancet, Jan. 21st, 1837, (p. 587) an analogous case is reported by Mr. King. It is remarkable that in all these cases the anomaly has occurred on the right side, and mostly in women.—*London Lancet*, July 15, from *Schmidt's Jahrbuch*, vol. xxxvii., p. 312.

